# Involvement of Long Non-Coding RNAs in Glucose Metabolism in Cancer

**DOI:** 10.3390/cancers13050977

**Published:** 2021-02-26

**Authors:** Amar Balihodzic, Dominik A. Barth, Felix Prinz, Martin Pichler

**Affiliations:** 1 Research Unit of Non-Coding RNAs and Genome Editing, Division of Oncology, Department of Internal Medicine, Comprehensive Cancer Center Graz, Medical University of Graz, 8036 Graz, Austria; amar.balihodzic@protonmail.com (A.B.); dominik.barth@medunigraz.at (D.A.B.); felix.prinz@medunigraz.at (F.P.); 2 BioTechMed-Graz, 8010 Graz, Austria; 3 Department of Experimental Therapeutics, The University of Texas MD Anderson Cancer Center, Houston, TX 77030, USA

**Keywords:** long non-coding RNA, lncRNA, cancer, glucose metabolism

## Abstract

**Simple Summary:**

Long non-coding RNAs (lncRNAs) are a heterogenous group of transcripts that regulate various cellular functions. They are implicated in all hallmarks of cancer, including metabolic alterations. Through the modulation of expression of oncogenic or tumor-suppressive genes, alteration of various signaling pathways, protein stability, and upregulation of metabolic enzymes, lncRNAs enhance glucose uptake in cancer and, thus, favor cancer progression. These transcripts represent crucial regulators of cancer glucose metabolism and, as such, they are potential clinical biomarkers and therapeutic targets. This review aims to provide an overview of the lncRNAs involved in cancer glucose metabolism and summarizes their underlying molecular mechanisms.

**Abstract:**

The rapid and uncontrolled proliferation of cancer cells is supported by metabolic reprogramming. Altered glucose metabolism supports cancer growth and progression. Compared with normal cells, cancer cells show increased glucose uptake, aerobic glycolysis and lactate production. Byproducts of adjusted glucose metabolism provide additional benefits supporting hallmark capabilities of cancer cells. Long non-coding RNAs (lncRNAs) are a heterogeneous group of transcripts of more than 200 nucleotides in length. They regulate numerous cellular processes, primarily through physical interaction with other molecules. Dysregulated lncRNAs are involved in all hallmarks of cancer including metabolic alterations. They may upregulate metabolic enzymes, modulate the expression of oncogenic or tumor-suppressive genes and disturb metabolic signaling pathways favoring cancer progression. Thus, lncRNAs are not only potential clinical biomarkers for cancer diagnostics and prediction but also possible therapeutic targets. This review summarizes the lncRNAs involved in cancer glucose metabolism and highlights their underlying molecular mechanisms.

## 1. Introduction

Cancers are characterized by uncontrolled and rapid cellular proliferation. To satisfy the accompanying increased demand for energy, cancer cells have to reprogram their metabolism, especially glucose metabolism [1,2]. Normal cells commonly acquire energy via glycolysis in the cytosol and oxidative phosphorylation in the mitochondria, which produces more ATP per molecule of glucose. When oxygen is deficient, cells primarily rely on glycolysis rather than on oxygen-consuming mitochondrial oxidative phosphorylation [3]. However, cancer cells favor glycolysis even in the presence of oxygen. This phenomenon is known as aerobic glycolysis or the Warburg effect [4]. Despite being known for decades, it was only recently acknowledged as a hallmark of cancer [5]. Cancer cells compensate for the net energy loss of glycolysis by an increased glucose uptake and eventual lactate production. Lactate lowers the surrounding pH, which additionally aids cancer invasion and metastasis, while glycolysis intermediates act as substrates that are used in the synthesis of anabolic components including nucleic acids, fatty acids, membrane phospholipids and proteins [3,6,7]. Compared with oxidative phosphorylation, glycolysis produces smaller amounts of reactive oxygen species, which can induce senescence and apoptosis of cancer cells [8]. Therefore, the Warburg effect provides great benefits to cancer thereby supporting its malignant features.

**Figure 1:**
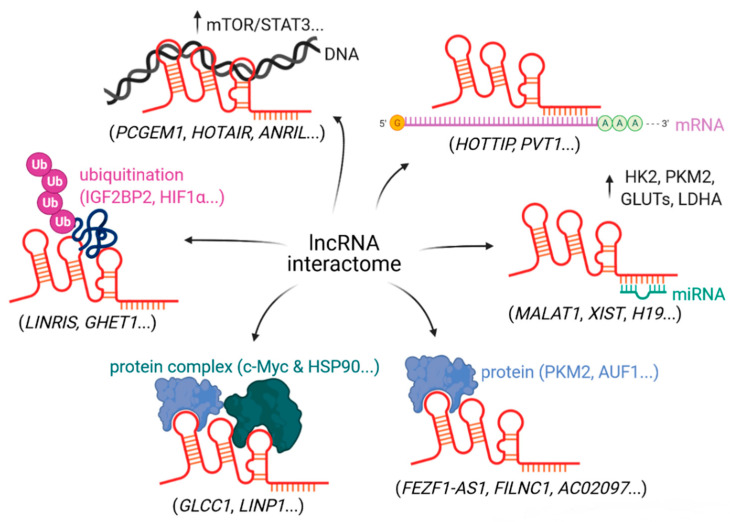
Schematic representation of a long non-coding RNA (lncRNA) interactome. LncRNAs interact with various molecules to perform important cellular functions (created with BioRender).

Long non-coding RNAs (lncRNAs) represent a heterogeneous group of RNA transcripts that are more than 200 nucleotides in length [9,10]. They comprise a large portion of the transcriptome but usually do not translate into proteins. However, lncRNAs share features with messenger RNA (mRNA) such as RNA polymerase-dependent transcription and post-transcriptional RNA processing including capping, splicing and polyadenylation [11,12]. LncRNAs exert important cellular functions through different molecular mechanisms, above all by interacting with various molecules [13]. By means of binding to DNA, RNA and protein, lncRNAs regulate gene expression via controlling the chromatin structure, methylation status, sequestration of miRNA, assembly or disruption of protein complexes including transcription factors and post-translational modifications such as phosphorylation and ubiquitination (Figure 1) [14,15,16]. Based on their genomic localization and connection to protein coding genes, lncRNAs can be classified as intergenic, intronic, enhancer, sense, antisense and bidirectional. Besides their genomic context, lncRNAs can also be categorized based on their functionality into signaling, guide, scaffold or decoy molecules [10,17]. Indeed, their functions are largely dependent on their subcellular localization [18].

**Figure 2:**
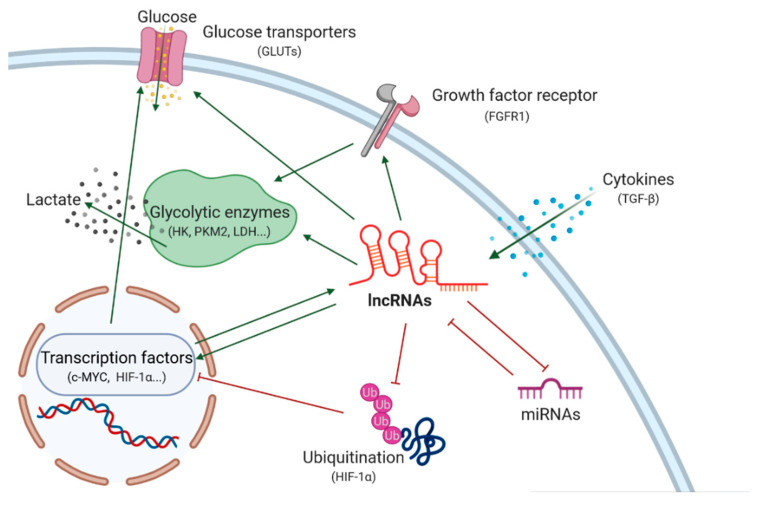
Schematic representation of glucose metabolism regulation in cancer by lncRNAs (created with BioRender.com).

Multiple studies have documented an aberrant lncRNA expression in various cancers where they act as oncogenes or tumor suppressors [19,20,21]. A few of these contributions to cancers are induced by lncRNAs via their ability to regulate glucose metabolism. They upregulate metabolic enzymes, disturb metabolic signaling pathways and modulate the expression of oncogenic or tumor-suppressive genes (Figure 2). In addition to potentially being useful as clinical biomarkers for cancer diagnostics and prediction, lncRNAs are also increasingly being considered as potential therapeutic targets [22]. 

The aim of this review is to provide an overview of the lncRNAs involved in cancer glucose metabolism. Additionally, we summarize the molecular mechanisms by which they exert these effects.

## 2. Methods

This review article is based on a literature review using the PubMed database. The following terms were used to search for the articles: ‘glucose metabolism’, ‘long-non-coding RNA’, ‘lncRNA’, ‘cancer’. Cancer studies in which the involvement of lncRNA in glucose metabolism was confirmed were used for further evaluation and were incorporated into this review. 

## 3. Results

A total of 63 articles meeting the entry criteria is included in this review article. The main findings are interpreted through two main sections. The first section summarizes the pathophysiological processes of cancer regulated by lncRNAs and their influence on glucose metabolism. The second section summarizes the general findings according to the types of cancers.

### 3.1. LncRNAs Regulates Pathophysiological Processes in Cancer Via the Influence of Glucose Metabolism

LncRNAs are dysregulated in various pathophysiological processes. A few of these processes include interactions with the tumor microenvironment and drug resistance. 

Numerous studies have documented altered expression of various lncRNAs under hypoxic conditions. Hypoxic stress, in turn, increases the rates of glucose consumption and lactate production [23,24]. For example, lncRNA *AC020978* is upregulated in non-small cell lung cancer (NSCLC) under glucose deprivation and hypoxia [25]. Similarly, the lncRNA HOXA transcript at the distal tip (*HOTTIP*) promotes glycolysis under hypoxic conditions via the miR-615-3p/HMGB3 axis in NSCLC cells [26]. In ovarian cancer, gastric carcinoma high expressed transcript 1 (*GHET1*) interacts with the E3 ubiquitin ligase von Hippel-Lindau (VHL). This consequently prevents the VHL-mediated degradation of HIF1α. Increased levels of HIF1α promote the glucose uptake and lactate production in ovarian cancer cells [27]. In addition, the HIF1α/lncRNA Retinoic Acid Early Transcript 1K (*RAET1K*)/miR-100-5p axis modulates hypoxia-induced glycolysis in hepatocellular carcinoma (HCC) cells and affects HCC progression [28].

Moreover, extracellular cytokines may increase the expression of lncRNAs. Urothelial carcinoma-associated 1 (*UCA1*) is upregulated by TGF-β1 in HCC. *UCA1* then upregulates metabolic enzymes and increases glucose uptake and lactate production [29]. Interestingly, even microorganisms may modify glucose metabolism in cancer via lncRNAs. *Fusobacterium nucleatum*, a member of the normal flora in the oral cavity, activates lncRNA *ENO1-IT1* transcription that eventually induces glucose metabolism in colorectal cancer (CRC) [30].

Furthermore, drug resistance is one of the biggest challenges in cancer treatment. Understanding the contributing factors is of the utmost importance in achieving better clinical outcomes [31]. Emerging studies correlate many lncRNAs with drug resistance across different types of cancers. *UCA1* is upregulated in acute myeloid leukemia (AML) following adriamycin (ADR)-based chemotherapy. It contributes to HIF1α-dependent glycolysis in AML [32]. This lncRNA is also upregulated in radioresistant cervical cancer cell lines and promotes glycolysis. In cytarabine-resistant AML cell lines, HOX antisense intergenic RNA myeloid 1 (*HOTAIRM1)* increases glucose consumption, lactate production and cellular proliferation [33]. In addition, lncRNA *H19* overexpression in ginsenoside 20(S)-Rg3-treated ovarian cancer cells increases glucose consumption, lactate production and pyruvate kinase isozyme M2 (PKM2) expression [34]. Another study proved increased glucose consumption and lactate secretion in cetuximab-resistant CRC cells, suggesting that glucose metabolism might be involved in cetuximab resistance. In the resistant cell lines, *LINC00973* was upregulated [35].

### 3.2. LncRNAs and Glucose Metabolism in Different Cancer Entities

#### 3.2.1. Gastrointestinal Cancers

Gastrointestinal cancers rank among the most commonly diagnosed cancers worldwide. In 2018, almost six million new cases were reported and around four million people died [36]. Moreover, these cancers are among the most commonly studied for the roles of lncRNAs [37,38,39]. The gastrointestinal tract comprises the organs that mediate digestion, suggesting that altered glucose metabolism may be an important factor in the development and progression of cancers of this system [40]. See Table 1 for a summary of lncRNA involvement in glucose metabolism in gastrointestinal cancers. 

##### Colorectal Cancer

A common lncRNA-induced disarragement of glucose metabolism in colorectal cancer (CRC) is mediated via the *c-MYC* oncogene. Many genes that are important for cell growth and cancer progression are targeted by *c-MYC* including those that regulate metabolism [41]. Glycolysis-associated lncRNA of colorectal cancer (*GLCC1*), also referred to as *AF339830*, is an oncogenic lncRNA in CRC. It is associated with poor overall survival (OS) of CRC patients (*p* = 0.0023). A higher *GLCC1* expression leads to increased celllular proliferation and an upregulated glycolysis pathway while experimental downregulation results in a significant reduction of cellular proliferation and tumor growth in mouse models. In addition, *GLCC1* stabilizes *c-MYC* and prevents its degradation by combining it with a heat shock protein 90 (HSP90) chaperon. This further increases the transcriptional level of lactate dehydrogenase A (LDHA), which then activates glycolytic metabolism. It has also been found that glucose starvation significantly increases *GLCC1* expression, suggesting that *GLCC1* may be a glucose starvation-induced oncogenic lncRNA in CRC [42]. The same physicial interaction with *c-MYC* and resulting effects have been observed for *LINC00504* [43]. Similarly, oncogenic long intergenic non-coding RNA for IGF2BP2 Stability (*LINRIS*) is important for glycolysis maintainance in CRC. It interacts with insulin-like growth factor 2 mRNA-binding protein 2 (IGF2BP2) and blocks its degradation through the ubiquitination-autophagic pathway. In turn, *c-MYC*, a downstream target of IGF2BP2, is stabilized. Consequently, the expression of MYC-related metabolic enzymes glucose trasporter 1 (GLUT-1), pyruvate kinase isozyme M2 (PKM2) and LDHA is increased [44]. 

In contrast, tumor-suppressive lncRNA RAD51 Antisense RNA1 (*RAD51-AS1*) regulates CRC progression by acting as a competing endogenous RNA (ceRNA) for miR-29b-3p and miR-29c-3p. As a consequence of their upregulation, their common target N-myc downstream-regulated gene 2 (*NDRG2*) is upregulated. Eventually, this leads to increased levels of hexokinase 2 (HK2) and GLUT-1 as well as elevated glucose consumption and lactate production. Interestingly, *RAD51-AS1* expression is not significantly associated with a worse OS of patients (logrank *p* = 0.35). However, decreased *NDRG2* expression is associated with a poor OS (logrank *p* = 0.043) [45]. A newly identified oncogenic lncRNA in renal cancer [46,47], lncRNA Activated in RCC with Sunitinib Resistance (*lncARSR*), is also highly expressed in CRC tissues and predicts poor disease-free survival (DFS) (hazard ratio (HR), 2.484; 95% CI, 1.132–6.232; *p* = 0.028) and OS (HR, 3.250; 95% CI, 1.596–6.716; *p* = 0.001). In the molecular context, *lncARSR* sponges miR-34a-5p and, consequently, enhances hexokinase 1 (HK1)-mediated aerobic glycolysis in vitro and in vivo [48].

Wu and colleagues [35] investigated the underlying differences between cetuximab-resistant and cetuximab-sensitive CRC cells. They proved an increased glucose consumption and lactate secretion in cetuximab-resistant cells, suggesting that glucose metabolism might be involved in cetuximab resistance. In addition, lncRNA *LINC00973* was upregulated in the resistant cell lines. Upon siRNA-mediated *LINC00973* downregulation, the resistant cells exhibited increased apoptosis, reduced cell viability and decreased glucose consumption and lactate production [35].

Huang et al. [49] confirmed the upregulation of lncRNA FEZF1 antisense RNA 1 (*FEZF1-AS1*) in two expanded CRC cohorts. They revealed that *FEZF1-AS1* expression is associated with CRC cell proliferation, metastasis and a poor OS (logrank *p* = 0.004) and DFS (logrank *p* = 0.019). Moreover, they found that *FEZF1-AS1* binds to cytoplasmic and nuclear PKM2 protein thereby increasing its stability and promoting its activity. Cytoplasmic PKM2 promotes pyruvate kinase activity and lactate production (aerobic glycolysis), whereas nuclear PKM2 acts as a protein kinase and activates STAT3 signaling. A multivariate analysis showed that *FEZF1-AS1* expression is an independent risk factor for the survival of patients with CRC (HR, 2.240; 95% CI, 1.028–4.878; *p* = 0.042) [49].

A recent study found that microbiota might interact with lncRNAs to modulate glucose metabolism in CRC. Specifically, *Fusobacterium nucleatum* (*F. nucleatum*) induces glucose metabolism and colorectal carcinogenesis via lncRNA enolase1-intronic transcript 1 (*ENO1-IT1*). *F. nucleatum* activates lncRNA *ENO1-IT1* transcription by increasing the binding efficiency of the transcription factor specificity protein 1 (SP1) to the promoter region of *ENO1-IT1*. Upregulated *ENO1-IT1* mediates the histone modification of several genes including the alpha-enolase glycolytic enzyme ENO1 via KAT7 histone acetyltransferase [30]. 

LncRNA colorectal neoplasia differentially expressed (*CRNDE*) is upregulated in CRC. Treatment with insulin and insulin-like growth factors (IGF) represses *CRNDE* nuclear transcripts. However, inhibitors against the PI3K/Akt/mTOR or Raf/MAPK pathway have opposite effects, suggesting that *CRNDE* is a downstream target of these signaling pathways. Additionally, *CRNDE* promotes metabolic changes in CRC mainly by upregulating the genes responsible for glucose-6-phosphate anabolic reactions (*GYS1*, *G6PC3*, *ISYNA1* and *G6PD*). *CRNDE* also positively regulates GLUT-4 and carbohydrate-responsive element-binding protein (MLXIPL) levels, leading to an increased glucose uptake and lactate production [50].

##### Hepatocellular Carcinoma

Liver-specific cytoplasmic long intergenic non-coding RNA *LINC01554* is downregulated in hepatocellular carcinoma (HCC). The downregulation of this tumor-suppressive lncRNA is associated with adjacent organ invasion, tumor size, advanced tumor stage and an overall poor outcome in patients with HCC [65]. *LINC01554* promotes proteasomal degradation of PKM2, an important enzyme at the late stage of glycolysis, and inhibits the Akt/mTOR signaling pathway, one of the key signalling pathways that mediates cellular biosynthesis and aerobic glycolysis in cancer cells. Mechanistically, *LINC01554* expression is inhibited by miR-365a-3p at the transcriptional level [51]. The effect of lncRNA metastasis-associated lung adenocarcinoma transcript 1 (*MALAT1*) on glucose metabolism is essential for its oncogenic activity in HCC. It upregulates the metabolic transcription factor TCF7L2 via the mTORC1 pathway, which then enhances the expression of glycolytic genes and downregulates enzymes involved in gluconeogenesis. In HCC, gluconeogenesis inhibits aerobic glycolysis and has a tumor-suppressive role. *MALAT1* overexpression results in a higher glucose uptake and increased lactate production [52]. HOX transcript antisense RNA (*HOTAIR*) is another upregulated lncRNA in HCC that activates the mTOR signaling pathway. It promotes glycolysis and lactate production by upregulating and directly binding to GLUT-1 [55]. Additionally, *HOTAIR* might act as a decoy of miR-130a-3p, which normally targets HIF1α [56].

Wang and colleagues [28] demonstrated that the HIF1α/lncRNA Retinoic Acid Early Transcript 1K (*RAET1K*)/miR-100-5p axis modulates hypoxia-induced glycolysis in HCC cells and affects HCC progression. The expression of lncRNA *RAET1K*, HIF1α and LDHA is upregulated in HCC tissue specimens while the miR-100-5p expression level is decreased. Mechanistically, *RAET1K* downregulates the expression of miR-100-5p by acting as a sponge while HIF1α binds the promoter region of the *RAET1K* and activate its transcription. *RAET1K* silencing significantly suppressed HCC cell proliferation and invasion. It also suppressed the hypoxia-induced increase in lactate concentration and glucose uptake while miR-100-5p inhibition reversed the effects of *RAET1K* silencing on hypoxia-induced glycolysis in HCC cells [28]. Upregulated lncRNA five prime to Xist (*Ftx*) is associated with aggressive clinicopathological features of HCC. In addition, *Ftx* enhances the activity and expression of key enzymes in glucose metabolism GLUT-1 and GLUT-4. Mechanistically, *Ftx* directly regulates the transcriptional and post-transcriptional expression of PPARγ. The PPARγ pathway is responsible for the *Ftx*-induced aerobic glycolysis in HCC [53].

It has been shown that TGF-β1 upregulates the expression of lncRNA urothelial carcinoma-associated 1 (*UCA1*) in HCC, which in turn significantly increases the expression level of HK2. Consequently, lactate production, glucose uptake and ATP production are increased while the OS is decreased (*p* = 0.0152) [29]. LncRNA *SOX2OT* promotes glucose metabolism by PKM2, HK2 and LDHA activation and increases the metastatic potential of HCC. *SOX2OT* physically binds to miR-122-5p, which also has a PKM2 targeting site and contributes to the PKM2 induction [58]. LncRNA DEAD/H box protein 11 antisense RNA 1 (*DDX11-AS1*) directly binds to tumor-suppressive miR-195-5p, thus allowing *MACC1*, a key regulator of the hepatocyte growth factor-HGF receptor (HGFR) pathway, to be overexpressed in HCC. As a result of *DDX11-AS1* overexpression, glucose consumption, lactate production, cellular proliferation, invasion and migration are increased [57].

Yu and colleagues [54] investigated the correlation between HCC and blood glucose dysregulation. HCC cells treated with a high glucose concentration were shown to have an increased expression of miR-483-3p and a decreased expression of lncRNA maternally expressed gene 3 (*MEG3*). Both of them led to suppressed endoplasmic reticulum protein 29 (ERp29) expression, which resulted in an increased HCC cell proliferation and migration. They found that *MEG3* mediates protective effects via binding to miR-483-3p. When *MEG3* is downregulated, overexpressed miR-483-3p suppresses ERp29 expression. Normally, ERp29 decreases cell proliferation and prevents epithelial-mesenchymal transition [54].

##### Gastric Cancer

Xia et al. [59] investigated the role of lncRNA DLX6 antisense RNA 1 (*DLX6-AS1*) in gastric cancer. *DLX6-AS1* exhibits oncogenic effects on multiple cancers including gastric cancer [66,67,68,69,70,71,72,73]. A higher *DLX6-AS1* expression is positively correlated with tumor size, lymph node involvement and tumor-node-metastasis (TNM) staging. In the molecular context, this lncRNA downregulates miR-4290, which targets 3-phosphoinositide-dependent protein kinase 1 (PDK1). PDK1 upregulation significantly increases intracellular levels of glucose uptake, ATP and lactate production. Aerobic glycolysis was efficiently elevated by PDK1 overexpression while the mitochondrial respiration was inhibited [59]. In addition, lncRNA DiGeorge syndrome critical region gene 9 (*DGCR9*) has an increased expression in gastric cancer. *DGCR9* increases cellular proliferation, migration and glucose uptake. *DGCR9* is positively associated with lymph node invasion and the TNM stage in gastric cancer patients [60].

##### Pancreatic Cancer

LncRNA *HOTAIR* is overexpressed in patients with pancreatic adenocarcinoma. *HOTAIR* alters cancer cell energy metabolism in pancreatic adenocarcinoma by the upregulation of HK2. This leads to the enhancement of tumor cell proliferation, glucose uptake, increased ATP and lactate production [61].

##### Esophageal Cancer

Zhao et al. [62] investigated the function of long intergenic non-protein coding RNA 184 (*LINC00184*) on glycolysis and mitochondrial oxidative phosphorylation of esophageal cancer cells. They found that highly expressed *LINC00184* increases esophageal cancer cell proliferation, migration, invasion and colony formation. *LINC00184* recruits DNA methyltransferase 1 (DNMT1) to tumor-suppressive phosphatase and the tensin homolog (PTEN) promotor region. Silenced PTEN, via Akt phosphorylation, leads to increased glycolysis and decreased mitochondrial oxidative phosphorylation [62]. Another lncRNA that was found to play a role in esophageal cancer is the lncRNA *UCA1*. *UCA1* mediates oncogenic effects by regulating glucose metabolism in esophageal cancer. It significantly suppresses the degradation of HK2 by sponging miR-203. Upregulated *UCA1* increases the glucose uptake, lactate output and extracellular acidification rate (ECAR) value [63].

##### Gallbladder Cancer

Sun and colleagues [64] reported that lncRNA plasmacytoma variant translocation 1 (*PVT1*) acts as an oncogenic lncRNA in gallbladder cancer (GBC) by sponging and repressing miR-143, allowing HK2 to be overexpressed. The upregulation of HK2 is responsible for the higher cellular glucose consumption and lactate production, which gives GBC cells an edge for proliferation, migration and invasion [64]. In addition, *PVT1* has been found to be overexpressed in multiple other cancers including CRC, non-small cell lung cancer (NSCLC) and pancreatic cancer [74,75,76]. However, its implications in glucose metabolism disruption in these cancer entities has yet to be investigated.

#### 3.2.2. Genitourinary Cancers

Genitourinary (GU) cancers comprise of a group of cancers in urinary and male reproductive systems [77]. They affect a large number of patients each year with prostate cancer being the most commonly diagnosed cancer and the second leading cause of death in males [78]. Table 2 provides and overview of lncRNA involvement in glucose metabolism in genitourinary cancers.

##### Kidney Cancer

Kidney-specific lncRNA FoxO-induced long non-coding RNA 1 (*FILNC1*) is downregulated in renal cell carcinoma correlating with poor clinical outcomes. Upon glucose starvation, the transcription factor FoxO upregulates *FILNC1* expression. In turn, *FILNC1* interacts with *c-MYC* mRNA-binding protein (AUF1), which prevents the interaction of AUF1 with *c-MYC* mRNA. This leads to the downregulation of *c-MYC* protein levels. However, in renal cancer, this pathway is altered. *FILNC1* is downregulated leading to increased *c-MYC* protein levels thereby increasing the glucose uptake, lactate production and tumor development [79].

##### Bladder Cancer

Duan et al. [80] observed that the lncRNA cancer susceptibility candidate 8 (*CASC8*) is significantly downregulated in bladder cancer and established its association with an advanced stage of bladder cancer. Normally, *CASC8* binds to the fibroblast growth factor receptor 1 (FGFR1), which in turn prevents FGFR1-mediated LDHA phosphorylation. Consequently, the conversion of pyruvate into lactate is attenuated, glycolysis is reduced and bladder cancer cell proliferation is slowed down [80]. As already observed in HCC and esophageal cancers, lncRNA *UCA1* also promotes glycolysis in bladder cancer cells. It regulates glycolysis by upregulating HK2 expression through mTOR and its downstream effector STAT3, which is a direct transcriptional activator for HK2 [81]. An additional mechanism of positive HK2 regulation is mediated through miR-143 downregulation by the active mTOR [82].

##### Prostate Cancer

Prostate cancer gene expression marker 1 (*PCGEM1*), a lncRNA highly associated with prostate tumors, significantly induces genes involved in multiple metabolic pathways such as aerobic glycolysis, pentose phosphate shunt and NADPH generation used for redox homeostasis. Mechanistically, *PCGEM1* binds directly to the target promoter of *c-MYC* where it functions as a coactivator and mediates enhanced *c-MYC* transcription [83]. 

#### 3.2.3. Female Reproductive System Cancers

##### Ovarian Cancer

Sood et al. [84] investigated the role of lncRNA ceruloplasmin (*NRCP*) in ovarian cancer metabolism. They found that *NRCP* is highly upregulated in ovarian tumors while its experimental knockdown leads to a decreased glycolysis and an increased mitochondrial respiration in cancer cells (Table 3). This, in turn, results in significantly increased cancer cell apoptosis and decreased cell proliferation. Mechanistically, *NRCP* acts as an intermediate binder between STAT1 and RNA polymerase II thereby increasing their activities. This leads to an elevated expression of the downstream target genes glucose-6-phosphate isomerase (*GPI*), aldolase A (*ALDOA*) and aldolase C (*ALDOC*) [84]. Furthermore, lncRNA gastric carcinoma high expressed transcript 1 (*GHET1*) is overexpressed in ovarian cancers and it is positively correlated with the tumor size and metastasis in patients. The overexpression of *GEHT1* promotes proliferation and colony formation of ovarian cancer cells. *GHET1* interacts with the E3 ubiquitin ligase von Hippel-Lindau (VHL), which consequently prevents the VHL-mediated degradation of HIF1α. Increased levels of HIF1α promote the glucose uptake and lactate production of ovarian cancer cells [27]. The important roles of lncRNAs in the regulation of HIF1α in cancer have been demonstrated previously [23]. It was observed that lncRNA *H19* overexpression in ginsenoside 20(S)-Rg3-treated ovarian cancer cells enhances glucose consumption, lactate production and PKM2 expression. *H19* directly sponges miR-324-5p to enhance the Warburg effect. Unaffected miR-324-5p inhibits PKM2 and negatively regulates glucose uptake and lactate production in ovarian cancer cells and inhibits cell proliferation [34].

##### Cervical Cancer

Yao and colleagues [85] showed that lncRNA *UCA1* has increased expression levels in radioresistant cervical cancer cell lines and promotes glycolysis. In addition, they found that this mechanism is regulated by the HK2 enzyme. These findings suggest that *UCA1* plays an important role in the regulation of radioresistance in cervical cancer via the HK2/glycolytic pathway [85].

#### 3.2.4. Breast Cancer

Lin and colleagues [86] demonstrated that lncRNA LINC00538 (*YIYA*) promotes cancer glycolysis, cell proliferation and tumor growth in breast cancer (Table 4).

Mechanistically, *YIYA* associates with the cytosolic cyclin-dependent kinase CDK6. It regulates the CDK6-dependent phosphorylation of the fructose bisphosphatase PFK2 (PFKFB3) in a cell cycle-independent manner and the catalysis of glucose 6-phosphate to fructose-2,6-bisphosphate/fructose-1,6-bisphosphate. Both lncRNA *YIYA* and CDK6 are required to maintain enhanced glycolysis in breast cancer [86]. Another study proved that upregulated lncRNA breast cancer anti-estrogen resistance 4 (*BCAR4*) in triple negative breast cancer is transcriptionally targeted by Yes-associated protein (YAP) and it is required for YAP-promoted glycolysis through GLI2-dependent Hedgehog signaling. YAP is normally inhibited by the tumor-suppressive Hippo pathway, which is commonly disregulated in many cancers [87]. *BCAR4* associates with the Hedgehog signalling effector GLI2 and promotes the transcription of glycolytic activators HK2 and PFKFB3. The individual or simulanteous upregulation of *BCAR4* and GLI2 results in increased glucose uptake and lactate production. The overexpression of YAP (*p* = 0.0166) and *BCAR4* (*p* = 0.0173) correlates with a poor recurrence-free survival (RFS) for breast cancer patients [88]. *LINC00346* is also overexpressed in breast cancer cells. Its experimental knockdown results in reduced cell proliferation and glycolysis as well as induced apoptosis through the upregulation of miR-148a/b, which is a direct target of *LINC00346*. The lower glycolysis rate is due to a repressed GLUT-1 expression [89].

#### 3.2.5. Hematologic Cancer

Wang et al. [90] found that lncRNA antisense non-coding RNA at the INK4 locus (*ANRIL*) is overexpressed in acute myeloid leukemia (AML) patients and promotes disease development and progression via the modulation of glucose metabolism. This lncRNA is commonly involved in multiple solid tumors [91] (Table 5). The authors found that *ANRIL* increases AML cell survival via the AdipoR1 pathway, which is an important regulator in cell senescence and metabolism including the Warburg effect. *ANRIL* and AdipoR1 upregulation results in the increased expression of GLUT-1, which facilitates the transportation of glucose across the plasma membrane, and LDHA, the enzyme that converts pyruvate to lactate. In addition, AdipoR1 upregulates AMPK and SIRT1, the main regulators of cell senescence and cell metabolism. Thus, they are important targets in AML treatment [90]. Moreover, lncRNA in non-homologous end joining (NHEJ) pathway 1 (*LINP1*) is significantly overexpressed in AML patients. It promotes the malignant phenotype of AML cells and stimulates glucose metabolism [92]. Previous studies have confirmed that *LINP1* functions as a scaffold for interaction with several important regulators in NHEJ-mediated DNA repair [93,94]. *LINP1* upregulates the expression levels of GLUT-1 and LDHA by upregulating HNF4α, which in turn activates the AMPK/WNT5A signaling pathway. The knockdown of *LINP1* expression remarkably suppresses the glucose uptake and AML cell maintenance [92]. Oncogenic lncRNA *UCA1* is also upregulated in AML following adriamycin (ADR)-based chemotherapy. Mechanistically, *UCA1* acts as a ceRNA of miR-125a and directly binds to it. Consequently, HK2, a target of miR-125a, is upregulated while the miR-125-mediated inhibition of a HIF1α-dependent glycolysis is blocked [32]. HOX antisense intergenic RNA myeloid 1 (*HOTAIRM1*) is the lncRNA that is highly specific for maturing myeloid cells and is well-known for its implication in the development of AML [95,96]. In cytarabine-resistant AML cell lines, *HOTAIRM1* enhances cell viability, reduces apoptosis and enhances glucose consumption and lactate production. These effects are mediated via the activated Wnt/β-catenin pathway [33].

Wang et al. [97] found that lncRNA protein disulfide isomerase family A member 3 pseudogene 1 (*PDIA3P*) is highly expressed in multiple myeloma and correlates with the OS of patients. They found that *PDIA3P* regulates multiple myeloma growth and drug resistance through glucose 6-phosphate dehydrogenase (*G6PD*) and the pentose phosphate pathway (PPP). Specifically, *PDIA3P* interacts with *c-MYC* thereby enhancing its transactivation and binding to the *G6PD* promoter, thus stimulating *G6PD* expression and PPP flux [97]. Additionally, lncRNA *MALAT1* is upregulated in multiple myeloma serums and cells. It is responsible for increased cell viability, invasion and glycolysis. *MALAT1* sponges miR-1271-5p, inhibiting its functions. In turn, SRY-Box 13 (SOX13), a miR-1271-5p target, is upregulated [98]. SOX13 is a member of the SRY-related high-mobility group box (Sox) transcription factor family and is known to be involved in the progression of several tumors including glioma [99]. 

#### 3.2.6. Brain Cancers

Guo and colleagues [100] observed that the lncRNA X-inactive specific transcript (*XIST*) is upregulated in glioblastoma cell lines and tissue samples (Table 6). Knockdown experiments highlighted the importance of this lncRNA in glioblastoma cell viability, invasion, resistance to apoptosis and enhanced glucose metabolism. *XIST* acts as a ceRNA for miR-126. By sponging miR-126, insulin receptor substrate 1 (IRS1) is upregulated along with the PI3K/Akt pathway. In addition, protein levels of GLUT-1 and GLUT-3 receptors as well as cellular glucose uptake and lactate production are elevated [100]. This lncRNA is also involved in several other cancer entities including bladder cancer, cervical cancer and pancreatic cancer, always exerting its functions via sponging various miRNAs and thereby promoting cancer progression [101,102,103]. However, a potential mechanism of *XIST* in glucose metabolism disruption in these cancers is yet to be investigated.

Furthermore, overexpressed *LINC00174* leads to an increased cell proliferation, tumor growth, migration, invasion and glycolysis of glioma cells. This lncRNA interacts with the miR-152-3p/SLC2A1 axis. *LINC00174* accelerates glioma carcinogenesis via sponging miR-152-3p. Consequently, SLC2A1 (GLUT-1), a downstream target of miR-152-3p, is upregulated. SLC2A1 is a crucial transporter for glucose uptake and plays an important role in glycolysis [104]. LncRNA small nucleolar RNA host gene 9 (*SNHG9*) is also elevated in glioblastoma tissues and it is associated with a lower OS (*p* = 0.0106) and PFS (*p* = 0.0014) in patients with glioma. The overexpression of *SNHG9* increases the cell proliferation and aerobic glycolysis. Mechanistically, *SNHG9* downregulates miR-199a-5p, leading to the upregulation of the Wnt2 axis in glioblastoma cells [105]. The upregulation of lncRNA *SNHG5* in glioma positively correlates with glucose uptake, migration and invasion of glioma cells. By acting as a sponge for miR-205, which targets E2F transcription factor 3 (E2F3), *SNHG5* enhances glioma growth [106]. Similarly, overexpressed lncRNA *SNHG1* promotes glioma progression in vitro and in vivo. *SNHG1* regulates pleckstrin homology like domain family A member 1 (PHLDA1) expression by sponging miR-194, leading to an increased glioma cell glucose uptake, proliferation, migration, invasion, angiogenesis and in vivo tumor growth [107].

The expression level of lncRNA TP53 target 1 (*TP53TG1*) is significantly higher in human glioma tissues or cell lines compared with normal brain tissue. *TP53TG1* is shown to be important in cellular proliferation and migration. It also promotes the upregulation of several glucose metabolism related genes such as glucose-regulated protein 78 (*GRP78*), isocitrate dehydrogenase 1 (*IDH1*) and LDHA. Interestingly, these effects are more striking in situations of glucose deprivation than in high glucose conditions [108]. Similarly, increased levels of Metastasis Associated in Colon Cancer 1 Antisense RNA 1 (*MACC1-AS1*), particularly under glucose deprivation, result in increased cell viability and inhibited apoptosis. High levels of *MACC1-AS1* in glioma tissues and cells are associated with a poor prognosis of glioma patients (*p* = 0.0106). *MACC1-AS1* overexpression significantly increases the levels of GLUT-1, HK2, *G6PD*, MCT1, ATP, lactate and NAPDH. These effects of *MACC1-AS1* overexpression were found to be mediated via the AMPK pathway [109].

#### 3.2.7. Osteosarcoma

Yang and colleagues [110] investigated the oncogenic mechanisms of lncRNA taurine-upregulated gene 1 (*TUG1*) in osteosarcoma (Table 7). They reported inhibited glucose consumption, lactate production and cell viability of osteosarcoma cells upon *TUG1* knockdown. However, an increased expression of *TUG1* markedly increased the expression of HK2 [110]. Similarly, upregulated lncRNA Potassium Voltage-Gated Channel Subfamily Q Member 1 Overlapping Transcript 1 (*KCNQ1OT1*) facilitates proliferation and decreases the apoptosis of osteosarcoma cells. *KCNQ1OT1* contributes to the Warburg effect by stimulating *ALDOA* expression. *KCNQ1OT1* sponges miR-34c-5p, which directly targets and inhibits *ALDOA* expression [111]. Similar to its effect in GBC, *PVT1* upregulation has been observed in osteosarcoma where it is associated with a poor survival of patients (*p* = 0.011). Glucose uptake, lactate production and the expression of HK2 are all increased in osteosarcoma cells with an overexpression of *PVT1* and decreased by *PVT1* knockdown [112].

In contrast, lncRNA heart and neural crest derivatives expressed 2-antisense RNA 1 (*HAND2-AS1*) has a decreased expression in osteosarcoma compared with normal tissue and functions as a tumor suppressor. The disruption of lncRNA *HAND2-AS1* results in osteosarcoma cell proliferation through the alteration of glucose metabolism. It leads to an increased expression of GLUT-1, consequently enhancing glucose uptake and the proliferation of osteosarcoma cell lines. Interestingly, *HAND2-AS1* is not associated with metastasis formation [113]. Another study has shown that *HAND2-AS1* regulates osteosarcoma metabolism via sequestering fructose-1,6-bisphosphatase 1 (FBP1) from binding to HIF1α thereby increasing the HIF1α expression [114]. Additionally, a tumor-suppressive role of this lncRNA has recently been identified in endometrioid carcinoma that acts via different mechanisms [115]. The potential involvement in glucose metabolism alteration should be investigated. 

#### 3.2.8. Melanoma

*H19* acts as an oncogenic lncRNA in many human cancers [116,117]. Xu et al. [118] identified its molecular mechanisms in melanoma. They found that *H19* acts as a sponge for miR-106a-5p, leading to the upregulation of E2F3 transcription factor expression in melanoma cells. E2F3 promotes melanoma cell glucose metabolism and growth [118]. 

#### 3.2.9. Lung Cancer

Huang and colleagues [25] proved that lncRNA *AC020978*, which is upregulated in non-small cell lung cancers (NSCLC) (Table 8), has a significant correlation with advanced stage and poor clinical outcomes. *AC020978* promotes cell growth and induces metabolic reprogramming. It is upregulated under glucose deprivation and hypoxia. Mechanistically, *AC020978* directly interacts with PKM2 and enhances the stability of this protein, which is crucial for the Warburg effect. In addition, *AC020978* promotes the nuclear translocation of PKM2. It thereby acts as a protein kinase and interacts with many tumor-associated genes to accelerate carcinogenesis including HIF1α, *Oct-4*, STAT3 and *CTNNB1* that encode β-catenin [25]. Likewise, the lncRNA HOXA transcript at the distal tip (*HOTTIP*) promotes glycolysis under hypoxic conditions via the miR-615-3p/HMGB3 axis in NSCLC cells. Hypoxic stress increases the rates of glucose consumption and lactate production. Glycolysis rate is increased by the substantial increase of HK2 enzyme expression compared with control cells grown in a normoxic environment. In addition, hypoxia increases *HOTTIP* and high mobility group box 3 (HMGB3) expression while it suppresses miR-615-3p expression. Mechanistically, *HOTTIP* acts as a molecular sponge for miR-615-3p leading to an increased expression of its target HMGB3 [26]. HMGB3 is a well-known oncogenic member of chromatin-binding proteins that can regulate gene expression in various cancers via transcription factor binding modulation [119,120,121]. Moreover, lncRNA *MALAT1* is upregulated in NSCLC tissues and cells. It is responsible for enhanced cell proliferation, colony formation, reduced cell apoptosis in vitro and increased tumor growth in vivo. *MALAT1* knockdown decreases glucose consumption and lactate production. It mediates these effects by binding to miR-613 thereby inhibiting its interaction with copper metabolism MURR1 domain-containing 8 (COMMD8) [122]. Research on COMMD8 in cancers is scarce. However, in addition to this research, one more study has shown that the overexpression of COMMD8 mitigates malignant NSCLC features [123]. Another oncogenic lncRNA in NSCLC is brain cytoplasmic RNA 1 (*BCYRN1*). *BCYRN1* induces glycolysis and upregulates the expression levels of PKM2 in NSCLC cells. These effects are mediated by reducing the miR‑149 expression levels [124].

*LINC00551* has a low expression in lung adenocarcinoma and correlates with a worse OS of lung cancer patients (HR = 0.67, logrank *p* = 0.0014). It regulates the glycolytic pathway by suppressing glucose uptake and lactate production. Mechanistically, *LINC00551* inhibits glycolysis by impairing the *c-MYC*-mediated transcription of glycolytic enzymes particularly PKM2 [125].

#### 3.2.10. Head and Neck Cancer

LncRNA *ANRIL* is also highly expressed in patients with nasopharyngeal carcinoma (Table 9). Overexpression is associated with advanced stage cancer, a shorter OS (*p* = 0.027), DFS (*p* = 0.033), frequent recurrences, increased cell proliferation and transformation via a stem-like cancer cell induction. *ANRIL* reprograms glucose metabolism to meet the cancer’s increased needs for energy by increasing the glucose uptake. It does that by affecting the mTOR signal pathway, which in turn increases the expression of GLUT-1 and LDHA, essential genes in glucose metabolism [126].

Cheng and colleagues [127] identified a novel lncRNA *lnc-p23154* associated with oral squamous cell carcinoma (OSCC). The upregulation of *lnc-p23154* correlates with OSCC cell migration and invasion in vitro and in vivo. Additionally, they found that *lnc-p23154* is involved in OSCC glycolysis where it upregulates GLUT-1 expression. *Lnc-p23154* is primarily located in the nucleus and binds to the promoter region of miR-378a-3p, which represses GLUT-1 expression. Therefore, *lnc-p23154* plays an important role in GLUT-1-mediated glycolysis by inhibiting miR-378a-3p transcription and accelerating OSCC metastasis [127]. Moreover, lncRNA *UCA1* is upregulated in tongue squamous cell carcinoma (TSCC) and promotes proliferation, migration, invasion and glycolysis metabolism of TSCC cells. *UCA1* was suggested to sponge miR-138-5p whose downstream target is CCR7 [128].

## 4. Conclusions

To maintain the characteristics of uncontrolled and accelerated proliferation, cancer cells reprogram their metabolism. This provides cancer with numerous benefits enabling its growth and progression. As glucose is the main source of energy, it is excessively metabolized by cancer cells leading to increased lactate production. Byproducts of altered glucose metabolism are substrates essential for the building of new cellular components and they lead to a lower surrounding pH thereby aiding metastasis formation. Metabolic disarrangement is associated with poor outcomes in certain cancers. A few of the key players of glucose metabolism such as LDAH and PKM2 could be used in patient stratifications and the prediction of the disease outcomes.

LncRNAs are a heterogenous group of transcripts capable of mediating various cellular functions including the regulation of glucose metabolism. Different molecular mechanisms of lncRNAs have been described. Among the most common is the regulation of the expression of metabolic enzymes such as GLUTs, LDAH, PKM2 and HK2. Namely, *ANRIL*, *UCA1*, *HOTAIR* and *H19* are a few of the most familiar lncRNAs that target metabolic enzymes. In addition, lncRNAs may sponge to different miRNA and mRNA thus modulating the expression of numerous oncogenic or tumor-suppressive genes. Proteins such as *c-MYC* and HIF1α are frequently stabilized and/or upregulated whereas, in the same way, lncRNAs alter signaling pathways favoring cancer progression. For instance, mTOR/STAT3 is commonly dysregulated in various cancers.

In conclusion, this review highlights lncRNAs as crucial regulators of cancer glucose metabolism. A blockade of these mechanisms may alleviate cancer progression, therefore rendering lncRNAs a promising therapeutic target.

## Figures and Tables

**Table 1:** Examples of lncRNAs implicated in glucose metabolism alteration in gastrointestinal (GI) cancers.

lncRNA	Deregulation	Functions and Targets	Reference
*AF339830* (*GLCC1*)	Upregulated in CRC	Stabilizes *c-MYC*; upregulates LDHA; increases glycolysis	[42]
*LINRIS*	Upregulated in CRC	Prevents IGF2BP2 degradation; stabilizies *c-MYC*; upregulates GLUT-1, PKM2 and LDHA expession	[44]
*LINC00973*	Upregulated in cetuximab-resistant CRC cells	Increases glucose consumption and lactate production	[35]
*LINC00504*	Upregulated in colon cancer	Increases *c-MYC* transcriptional activity; increases glycolysis, fatty acid synthesis, maintains redox status	[43]
*FEZF1-AS1*	Upregulated in CRC	Stabilizes and promotes the activity of PKM2; upregulates glycolysis and STAT3 signaling	[49]
*ENO1-IT1*	*Fusobacterium nucleatum*-activated lncRNA in CRC	Upregulates the alpha-enolase glycolytic enzyme	[30]
*RAD51-AS1*	Downregulated in CRC	ceRNA for miR-29b-3p and miR-29c-3p; upregulates *NDRG2*; increases the levels of HK2 and GLUT-1, glucose uptake and lactate production	[45]
*lncARSR*	Upregulated in CRC	Sponges miR-34a-5p; upregulates HK1-mediated aerobic glycolysis	[48]
*CRNDE*	Upregulated in CRC	Upregulates *G6PD*, GLUT-4 and MLXIPL; increases glucose uptake and lactate production	[50]
*LINC01554*	Downregulated in HCC	Promotes PKM2 degradation; inhibits Akt/mTOR signaling	[51]
*MALAT1*	Upregulated in HCC	Upregulates TCF7L2 via the mTORC1 pathway; upregulates the expression of glycolytic enzymes	[52]
*Ftx*	Upregulated in HCC	Enhances the activity and expression of GLUT-1 and GLUT-4 via PPAR*γ*	[53]
*MEG3*	Downregulated in high glucose-treated HCC	Sponges miR-483-3p; suppresses ERp29 expression	[54]
*UCA1*	Upregulated in HCC	Increases the expression of HK2; enhances glucose uptake, lactate and ATP production	[29]
*RAET1K*	Upregulated in HCC	Sponges miR-100-5p; upregulates HIF1α; suppresses hypoxia-induced glucose uptake and lactate secretion	[28]
*HOTAIR*	Upregulated in HCC	Upregulates GLUT-1; activates mTOR signaling pathway; decoy for miR-130a-3p (HIF1α target); promotes glycolysis and lactate production	[55,56]
*DDX11-AS1*	Upregulated in HCC	Sponges miR-195-5p; increases *MACC1* expression; increases glucose consumption and lactate production	[57]
*SOX2OT*	Upregulated in HCC	Sponges miR-122-5p; activates PKM2, HK2 and LDHA	[58]
*DLX6-AS1*	Upregulated in gastric cancer	Sponges miR-4290; upregulates PDK1; elevates glycolysis rates; inhibits mitochondrial respiration	[59]
*DGCR9*	Upregulated in gastric cancer	Increases glucose uptake	[60]
*HOTAIR*	Upregulated in pancreatic cancer	Upregulates HK2; increases glucose uptake, ATP and lactate production	[61]
*LINC00184*	Upregulated in esophageal cancer	Silences PTEN; increases glycolysis and decreases mitochondrial oxidative phosphorylation	[62]
*UCA1*	Upregulated in esophageal cancer	Sponges miR-203; suppresses HK2 degradation; increases glucose uptake and lactate output	[63]
*PVT1*	Upregulated in gallbladder cancer	Sponges and repress miR-143; upregulates HK2; increases glucose uptake and lactate output	[64]

**Table 2:** Examples of lncRNAs implicated in glucose metabolism alteration in GU cancers.

lncRNA	Deregulation	Functions and Targets	Reference
*FILNC1*	Downregulated in renal cell cancer	Interacts with AUF1 (*c-MYC* mRNA-binding protein); downregulates *c-MYC* protein levels; decreases the glucose update, lactate production and tumor development	[79]
*CASC8*	Downregulated in bladder cancer	Binds to the fibroblast growth factor receptor 1 (FGFR1); prevents FGFR1-mediated lactate dehydrogenase A phosphorylation; decreases glycolysis and cell proliferation	[80]
*UCA1*	Upregulated in bladder cancer	Upregulates HK2 through mTOR and its downstream effector STAT3; upregulates HK2 by active mTOR downregulation of miR143	[81,82]
*PCGEM1*	Upregulated in prostate cancer	Binds to the *c-MYC* promotor; upregulates *c-MYC*; induces aerobic glycolysis, pentose phosphate shunt and NADPH	[83]

**Table 3:** Examples of lncRNAs implicated in glucose metabolism alteration in female reproductive system cancers.

lncRNA	Deregulation	Functions and Targets	Reference
*NRCP*	Upregulated in ovarian cancers	Acts as an intermediate binder between STAT1 and RNA polymerase II; increases the expression of glucose-6-phosphate isomerase (*GPI*), aldolase A (*ALDOA*) and aldolase C (*ALDOC*)	[84]
*GHET1*	Upregulated in ovarian cancers	Interacts with the E3 ubiquitin ligase von Hippel-Lindau (VHL); prevents VHL-mediated HIF1α-degradation; promotes glucose uptake and lactate production	[27]
*H19*	Upregulated in ginsenoside 20(S)-Rg3-treated ovarian cancers	Sponges miR-324-5p; upregulates PKM2; increases glucose uptake and lactate production	[34]
*UCA1*	Upregulated in radioresistant cervical cancer	Upregulates HK2	[85]

**Table 4:** Examples of lncRNAs implicated in glucose metabolism alteration in breast cancer.

lncRNA	Deregulation	Functions and Targets	Reference
*BCAR4*	Upregulated in triple negative breast cancer	Interacts with GLI2; upregulates HK2 and PFKFB3; increases glucose uptake and lactate production	[88]
*YIYA*	Upregulated in breast cancer	Interacts with CDK6; increases phosphorylation PFKFB3; catalysis of glucose 6-phosphate to fructose bisphosphate	[86]
*LINC00346*	Upregulated in breast cancer cells	Sponges miR-148a/b; enhances GLUT-1 expression	[89]

**Table 5:** Examples of lncRNAs implicated in glucose metabolism alteration in hematologic cancer.

lncRNA	Deregulation	Functions and Targets	Reference
*ANRIL*	Upregulated in acute myeloid leukemia (AML)	Increases the expression of GLUT-1 and LDAH, upregulates AdipoR1, AMPK and SIRT1	[90]
*LINP1*	Upregulated in AML	Increases the expression of GLUT-1 and LDHA; upregulates HNF4α; activates the AMPK/WNT5A signaling pathway	[92]
*UCA1*	Upregulated in AML	ceRNA of miR-125a; upregulates HK2; blocks HIF1α-dependent glycolysis	[32]
*HOTAIRM1*	Upregulated in AML	Activates the Wnt/β-catenin pathway; enhances glucose uptake and lactate production	[33]
*PDIA3P*	Upregulated in multiple myeloma	Interacts with *c-MYC*; upregulates *G6PD* expression and PPP flux	[97]
*MALAT1*	Upregulated in multiple myeloma	Targets miR-1271-5p; increases SOX13 expression	[98]

**Table 6:** Examples of lncRNAs implicated in glucose metabolism alteration in brain cancers.

lncRNA	Deregulation	Functions and Targets	Reference
*XIST*	Upregulated in glioblastoma	Sponges miRNA126; upregulates the IRS1 and PI3K/Akt pathways; upregulates the GLUT-1 and GLUT-3 receptors; increases cellular glucose uptake and lactate production	[100]
*LINC00174*	Upregulated in glioma	Sponges miR-1523-3p; upregulates SLC2A1	[104]
*SNHG9*	Upregulated in glioblastoma	Downregulates miR-199a-5p; upregulates the Wnt2 axis	[105]
*SNHG1*	Upregulated in glioma	Sponges miR-194; upregulates PHLDA1	[107]
*SNHG5*	Upregulated in glioma	Sponges miR-205; upregulates E2F transcription factor 3	[106]
*TP53TG1*	Upregulated in glioma	Upregulates *GRP78*, LDHA and *IDH1*	[108]
*MACC1-AS1*	Upregulated in glioma	Increases the levels of GLUT-1, HK2, *G6PD*, MCT1, ATP, lactate and NAPDH	[109]

**Table 7:** Examples of lncRNAs implicated in glucose metabolism alteration in osteosarcoma.

lncRNA	Deregulation	Functions and Targets	Reference
*TUG1*	Upregulated in osteosarcoma	Increases HK2 expression, glucose consumption, lactate production	[110]
*KCNQ1OT1*	Upregulated in osteosarcoma	Sponges miR-34c-5p; upregulates aldolase A (*ALDOA*) expression	[111]
*PVT1*	Upregulated in osteosarcoma	Increases HK2 expression, glucose consumption, lactate production	[112]
*HAND2-AS1*	Downregulated in osteosarcoma	Decreases GLUT-1 and glucose update	[113,114]

**Table 8:** Examples of lncRNAs implicated in glucose metabolism alteration in lung cancers.

lncRNA	Deregulation	Functions and Targets	Reference
*AC020978*	Upregulated in NSCLC	Interacts with and stabilizes PKM2; promotes PKM2 nuclear translocation; upregulates HIF1α, *Oct-4*, STAT3 and β-catenin	[25]
*HOTTIP*	Upregulated in NSCLC	Sponges miR-615-3p; increases HMGB3 expression; upregulates HK2 expression; increases glucose consumption and lactate production	[26]
*MALAT1*	Upregulated in NSCLC	Sponges miR-613; upregulates COMMD8; increases glucose consumption and lactate production	[122]
*BCYRN1*	Upregulated in NSCLC	Reduces miR‑149 expression; upregulates PKM2 expression; induces glycolysis	[124]
*LINC00551*	Downregulated in lung adenocarcinoma	Decreases *c-MYC* expression; decreases PKM2; inhibits glycolysis	[125]

**Table 9:** Examples of lncRNAs implicated in glucose metabolism alteration in head and neck cancer.

lncRNA	Deregulation	Functions and Targets	Reference
*ANRIL*	Upregulated in nasopharyngeal carcinoma	Upregulates mTOR signal pathway; increases GLUT-1 and LDHA expression	[126]
*lnc*-*p23154*	Upregulated in oral squamous cell carcinoma	Sponges miR-378a-3p; upregulates GLUT-1 expression	[127]
*UCA1*	Upregulated in tongue squamous cell carcinoma	Sponges miR-138-5p; upregulates CCR7	[128]
